# Literature Review on Ship Localization, Classification, and Detection Methods Based on Optical Sensors and Neural Networks

**DOI:** 10.3390/s22186879

**Published:** 2022-09-12

**Authors:** Eduardo Teixeira, Beatriz Araujo, Victor Costa, Samuel Mafra, Felipe Figueiredo

**Affiliations:** Instituto Nacional de Telecomunicações (INATEL), Santa Rita do Sapucaí 37540-000, MG, Brazil

**Keywords:** maritime surveillance, classification, localization, detection, artificial intelligence, neural networks

## Abstract

Object detection is a common application within the computer vision area. Its tasks include the classic challenges of object localization and classification. As a consequence, object detection is a challenging task. Furthermore, this technique is crucial for maritime applications since situational awareness can bring various benefits to surveillance systems. The literature presents various models to improve automatic target recognition and tracking capabilities that can be applied to and leverage maritime surveillance systems. Therefore, this paper reviews the available models focused on localization, classification, and detection. Moreover, it analyzes several works that apply the discussed models to the maritime surveillance scenario. Finally, it highlights the main opportunities and challenges, encouraging new research in this area.

## 1. Introduction

With the growth in ocean exploration by cruise ships, ocean liners, and other marine ships, the need for monitoring systems has increased considerably. With this, monitoring stations have become increasingly equipped to carry out the identification of possible issues. Among the maritime monitoring applications, one can mention potential collision prediction [1], navigation support, tracking of ships drift [2] target tracking, maritime safety [3]. Visual ship tracking provides crucial kinematic traffic information to maritime traffic participants, which helps to accurately predict ship traveling behaviors in the near future. Each of these applications requires different operating architectures [4].

Automatic maritime surveillance assumes the use of sensors that can provide enough information for automatic situational awareness tasks, such as localization and classification. In localization, a single object is found in an image. In classification, the object is defined as belonging to a specific class. Detection combines the characteristics of these two techniques to locate and classify multiple targets in the scene.

The fusion of different sensing sources can provide a better situational view of the monitored environment and help one take the necessary actions. Sensors based on sound or electromagnetic waves, such as sonar and radar, are generally employed in long-range applications. Optical sensors can be more economical alternatives for applications that require greater detail of the ships and aim for low power consumption. They can be employed in ship tracking and classification tasks, requiring only that these sensors be combined with visual detection techniques that are efficient, fast, and robust to enable the advancement of maritime applications [5].

The number of sensors involved can also vary, and the most common ones for this purpose are thermal cameras, optical cameras, and radar. Choosing the best techniques to obtain maritime situational information is not trivial since the literature offers a huge amount of model options that employ optical data. To make their application even more complicated, weather or water conditions, such as wind speed, tidal changes, rain, and fog, can, for example, blur or entirely obstruct objects in an image. Additionally, the increasing distance between the monitored object and the sensors can also aggravate visual tasks, as it can cause a high-scale variation.

At the beginning of the research on ship detection, such as the one on object detection in general, methods employing simple and handcrafted features were used, but recently, convolutional neural network (CNNs) have been added as part of this field of study because of their extraordinary ability to extract and represent visual features [6]. For example, in automatic navigation systems, the role of CNNs is to interpret the visual data collected by the cameras. Thus, the detection information is added to the data from different sensors, allowing the data fusion processing system to have enough information for decision-making.

The paper is organized as follows. Section 2 describes the theoretical background of the techniques, as well as presenting related works. Section 3 explores the different datasets found in the literature, detailing ship classes and image sample characteristics. Section 4 shows the challenges and open questions. Finally, Section 5 concludes this work with some concluding remarks and a discussion of future work, and outlines some lines of future research.

## 2. Related Works

In marine monitoring scenarios, localization, classification, and detection techniques are applied to data received from sensors to extract information on the location and type of the monitored ship. For example, in the case of optical sensors, commonly called cameras, an analysis of the images is performed within the viewing angle. In this case, images are received frame by frame and processed with the desired algorithm, be it for localization, classification, or detection.

**Figure 1:**
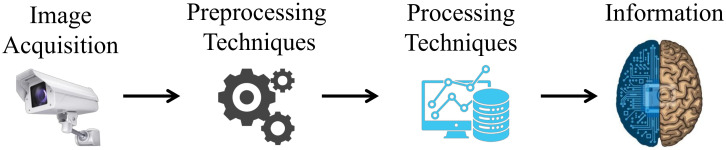
Components of an image processing system.

Several works are proposed by autonomous authors or those affiliated with some universities or research centers to perform the tasks in the localization, classification, and detection of ships [7]. Figure 1 shows a generic block diagram for optical sensor image processing systems. This system has four main stages: image acquisition, preprocessing, processing, and information output.

The first stage is responsible for frame capture, followed by signal preprocessing and the application of corresponding techniques for localization, classification, or detection. In the first stage, individual or joint optical sensors convert the light reflected by the objects into arrays of pixels representing the scene. The next stage, which may or may not exist depending on the system, is preprocessing. Here, the image is prepared for feature extraction using techniques for noise reduction, deblurring, feature intensification, and image quality enhancement [8].

The image enhancement process is usually applied so that the processing models perform better due to higher quality samples. With that, as long as the preprocessing is well implemented, the next step, such as extracting features or object segmentation, performs better [9]. Some jobs do not have a preprocessing stage, while others divide some of these stages into more than one task. The inclusion of the preprocessing step directly influences the parameters related to the performance of the models.

Among the factors that are influenced, it is possible to mention the total time of the image processing system and factors related to accuracy metrics. To calculate the factors related to the accuracy metrics, it is necessary to initially obtain four response parameters from these models: “true positive”, “true negative”, “false positive”, and “false negative”.

Once these parameters are obtained, it is possible to calculate the accuracy, which is the total number of correct predictions divided by the total number of predictions made for a dataset, and precision, which quantifies the number of positive class predictions that belong to the positive class. Some works also feature recall, which quantifies the number of positive class predictions made out of all positive examples in the dataset, and the F1-score, which provides a single score that balances the concerns of precision and recall in one number.

### 2.1. Image Acquisition

During image capture, several types of sensors can be used. However, the focus of this work is on the use of optical sensors, be they remote, installed on satellites or aircraft, or those that observe from a side view, such as those installed in inshore or offshore scenarios, such as on other ships or fixed constructions on land, usually near the coast.

Optical images can still be divided into visible and infrared (IR) spectra, and the range of both is very similar, from the order of meters to at most a few kilometers. The main differences between optical sensors are related to sensitivity to the environment and the quality and quantity of visual information generated by the sensor [7].

Comparing the sensitivity to illumination, both sensor types have problems working outside their respective designations, i.e., while the visible light sensor performs poorly for nighttime applications, the IR sensor presents high saturation in images captured during the day. In addition, the visible light sensor is less robust to the effects of light reflection on ships caused by water dynamics. However, the visual data generated are more detailed when compared to the quality and quantity of elements captured by a visible sensor [7]. Thus, this sensor can lead to the training of detectors with higher reliability.

Optical remote sensing images suffer from weather conditions, such as rain, waves, fog, and clouds, which causes the need, in some cases, for preprocessing of the image to improve the image quality that will be analyzed.

### 2.2. Preprocessing Techniques

The preprocessing step can be used to improve the quality of images by introducing techniques that allow obtaining a dataset with better quality images through the attenuation of interference caused by elements of the environment, such as extreme brightness and contrast, in addition to the quality of the camera lenses used in the capture process [10].

**Figure 2:**
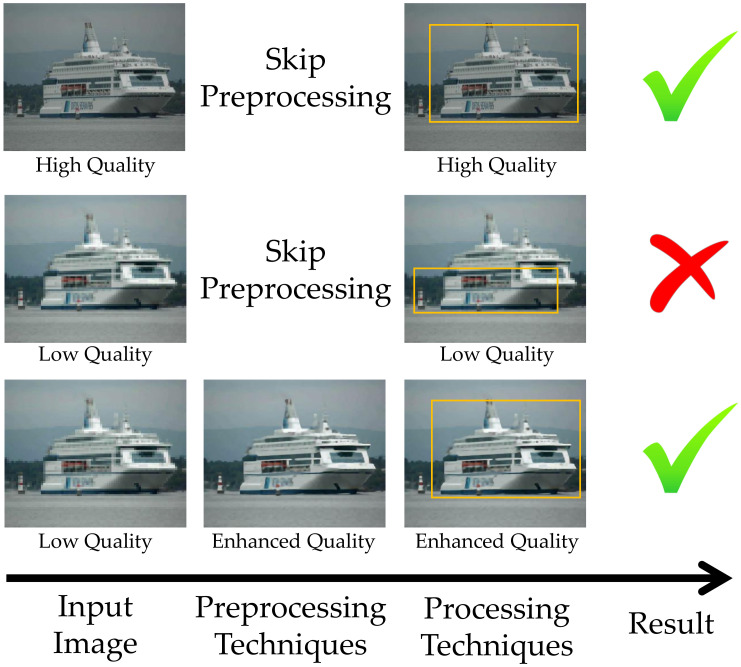
Detection enhancement with preprocessing.

Among the various techniques that can be used in preprocessing, it is possible to mention super-resolution techniques [11,12] and deblur [13,14]. The main benefit of improving images before they are used in location, classification, or detection models is usually the improvement of accuracy achieved only by increasing the quality of the dataset [15]. An example of detection enhancement can be seen in Figure 2.

Super-resolution techniques are used to recover quality and improve the resolution of an image. With this, instead of receiving low-resolution images, the model starts to operate with more detailed images, leading various situations to an instant performance improvement [16]. The field of image super-resolution has been dominated by methods based on convolutional neural networks in recent years [17]. Among the examples of models related to the super-resolution task, one can mention the models based on training to minimize the mean squared error (MSE), such as super-resolution convolutional neural network (SRCNN) [18], super-resolution residual network (SRResNet) [19], enhanced deep super-resolution network (EDSR) [20], multi-scale deep super-resolution (MDSR) [20], and deep back-projection networks (DBPN) [21], and also models based on generative adversarial network (GANs), such as super-resolution generative adversarial network (SRGAN) [22], enhanced super-resolution generative adversarial network (ESRGAN) [23], and rank super-resolution generative adversarial network (RankSRGAN) [24].

In addition to being based on different forms of training, these models also differ in the layer structures that make up their architectures, which influence their performance, both in aspects related to accuracy and their processing time. Super-resolution models trained through an MSE estimator use the distance between training images and associated predictions as a cost function. As a result, these models tend to produce smoother images. On the other, models based on GANs use generators to create new false images that can mimic the expected result so that the discriminative model has the maximum difficulty distinguishing between the synthesized images of the generator and the actual images. This process generates output images with more realistic detail but can cause some unwanted noise to enter the image during the super-resolution process [25].

#### 2.2.1. SRCNN

The SRCNN model is based on an CNN architecture, trained to learn an end-to-end mapping between low and high-resolution images for the super-resolution (SR) problem. It was one of the first architectures that applied the concept of deep learning in the super-resolution task, achieving one of the best results for this task in 2015. When the authors proposed the use of CNNs, the most common was to use the traditional sparse-coding-based SR methods [18]. The model is divided into three stages; the first is responsible for performing the extraction and representation of low-quality images within the network. The second step is the application of a nonlinear mapping, where the layers of CNN extract as much information about the image. The last step is to rebuild the image at a higher resolution than the image applied to the template entry [18].

#### 2.2.2. SRResNet

The model SRResNet was developed with an architecture based on residual network (ResNet), but with modifications in the optimization of the MSE loss function to achieve high upscaling factors, 4×, as quoted in [22]. With this optimization, the model was consolidated in 2017 as the new state-of-the-art, and its performance was evaluated by peak signal-to-noise ratio (PSNR) and structural similarity index measure (SSIM), two metrics widely used in image quality assessment [26].

Another proposal by the authors of [22] was to change the MSE loss function using model resources visual geometry group (VGG). With this, the authors compared the optimized version of the MSE with the version modified to use the VGG loss metrics. The result was that the model improved the visual metric mean opinion score (MOS) but had lower performance on the metrics of PSNR and SSIM [22].

#### 2.2.3. SRGAN

The model SRGAN is trained through a generative architecture based on the ResNet. This architecture generates images with super-resolution and has its loss analyzed through a second structure with a discriminative function, which only acts during training. The commission’s proposal SRGAN is to use a new resource-based loss function of the model VGG, which, when combined with the discriminating network, helps to detect the difference between the image generated about the reference image. According to metric-based tests MOS [27], which evaluates the perceptual quality of an empirically obtained image through a visual classification scale, the trained model achieves results close to the state of the art in the literature [22].

#### 2.2.4. EDSR

The model EDSR, as well as the other SR models already presented, also has its architecture based on ResNet. These models have characteristics similar to SRResNet. However, unnecessary modules are removed from the architecture to optimize the model. Among these changes, one can mention the residual blocks, which have removed the batch normalization (BN). This causes the model to be simplified and memory usage to be reduced [20]. Just as SRResNet achieves 4× upscaling factors, the EDSR model is also capable. In the work [20], the authors trained the model for upscaling of 2×, 3×, and 4×. In addition to achieving model time optimization and simplifying the architecture, the authors also had superior results compared to other networks that were tested, such as SRCNN, SRResNet, and MDSR.

#### 2.2.5. MDSR

The model MDSR was proposed by the same author who developed the EDSR model such that their architectures are described in [20]. The MDSR network has a certain increase in complexity compared to EDSR because it uses extra blocks with different scales at the beginning of the architecture. Removing the BN layers, as suggested in [20], is also adopted in this model [20]. Unlike EDSR, which reconstructs only a super-resolution image scale on the MDSR network, an initial upscaling is applied that operates with parallel image processing structures of different sizes. This allows for reducing various problems caused by variations in image scale. Both models, EDSR and MDSR, were proposed in the NTIRE 2017 Super-Resolution Challenge [28], taking first and second place, respectively. With this, the authors claimed that they managed simultaneously to achieve the state of the art in the topic of super-resolution and simultaneously transformed the architecture ResNet into a more compact model.

#### 2.2.6. ESRGAN

In three respects, the model ESRGAN is an improved version of the network SRGAN. The first was the replacement of residual blocks by residual-in-residual dense block (RRDB) to facilitate training, followed by the exchange of layers of BN by residual scaling and smaller initialization, as suggested in [20] because it allows the training of a more profound architecture. The second difference was the replacement of a GAN joint for a relativistic average GAN (RaGAN); instead of judging whether an image is true or false, this generative network can identify which image is more realistic. Finally, the perceptual loss was improved using VGG features before and after activation as in the SRGAN. This last change makes the model provide sharper edges and visually more satisfying results [23]. With the modifications made, the model reached the state of the art in 2018, presenting the best results of perceptual quality, with first place in the challenge perceptual image restoration and manipulation—super resolution (PIRM-SR) [17]. The test evaluated several models under the quality of visual perception metrics, PSNR and SSIM. From the evaluation of the performance of the models in this challenge, it was possible to notice that the increasing values of PSNR and SSIM were not always accompanied by an increase in perceptual quality. In many cases, this resulted in increasingly blurred and unnatural outputs, which gives more meaning to the previously cited results of [22].

#### 2.2.7. RankSRGAN

The RankSRGAN model is based on the GANs architecture but adopts a siamese architecture to learn perceptual metrics and rank images according to the quality score found during its training. This model combines different SR algorithms to improve perceptual metrics by combining other models [24]. To train the ranker, the authors used three templates, SRResNet, SRGAN, and ESRGAN. With their combination, RankSRGAN was able to optimize the natural image quality evaluator (NIQE) parameter [29], a visual metric that measures the naturalness of the image in the scene. With this, the model achieved superior performance to the individual models used when applied to the dataset of the PIRM-SR Challenge 2018 [24].

#### 2.2.8. DBPN

The DBPN model is an improved version of the SRCNN network, but instead of using predefined upsampling, it uses interleaved upsampling and downsampling layers. Unlike other methods that build the SR image feed-forwardly, our proposed networks focus on directly increasing SR resources by using multiple stages of ascending and descending sampling that feeds error predictions into each depth. The values of the error feedback of the steps of increase and scale reduction were used to guide the network to obtain a better result. The model performed similarly to the state-of-the-art performance in 2018. In addition, the network was trained with 8× magnification, higher than that used in the creation of SRResNet [19] and EDSR [20].

Unlike super-resolution techniques, deblurring techniques were developed to remove noise and blur present in the image, which hinders the visualization of the image. When noisy images are treated before being inserted into detection and classification systems, the system performance can increase considerably [30]. Some of the techniques that can be applied to the deblur task are deblur generative adversarial network (DeblurGAN), DeblurGAN-V2, and deblurring and shape recovery of fast moving objects (DeFMO).

#### 2.2.9. DeblurGAN

The DeblurGAN template is composed of a GAN architecture, and its purpose is to remove blur in images. The model features an architecture of CNN, composed of residual blocks (ResBlocks) consisting of a convolution layer, instance normalization layer, and ReLU activation [31]. The authors of DeblurGAN validated their results by applying the you only look once (YOLO) model to perform the detection and classification of objects in images with blur and images processed by the deblur model. There is a gain in accuracy in the YOLO results when inserted images are improved by the DeblurGAN model, proving that it significantly contributes to image quality and consequently to the performance of subsequent processing systems [31].

#### 2.2.10. DeblurGAN-V2

The DeblurGAN-V2 model is based on the construction of the original model DeblurGAN but with some modifications to improve the [32] network. Among them, the generative model in DeblurGAN-V2 integrates the technique feature pyramid network (FPN). This technique was initially developed for object detection purposes [33]. Still, in the case of the DeblurGAN-V2 model, the authors used FPN for the construction of a noisy image [32]. In addition to integrating the FPN technique, the new version allows the selection of different backbones. Each of the different backbones is designed to improve some of the performance parameters. For example, with the Inception-ResNetV2 architecture, you obtain a next-generation blur. In contrast, with the mobile network-depthwise separable convolution (MobileNet-DSC) architecture, you obtain an increase in processing speed, some 10 to 100 times faster than the top competitors in 2019 [32].

#### 2.2.11. DeFMO

Motion blur is one of the existing blur types, and it is caused by the rapid movement of objects when captured by cameras or by the quick scroll of the camera to capture still objects, recording photos or videos, with blur [34]. Thus, DeFMO is designed to act on this type of blur. The proposed network is a novel based on a ’self-supervised’ loss function that improves the model’s accuracy when applied to images with motion blur. By presenting a good generalization capability, this model can be applied to different areas in computer vision, such as the improvement of security cameras, microscopes, and photos with high noise levels [35]. This model is the first fully neural FMOs deblurring that fills the gap between deblurring, 3D modeling, and FMO subframe tracking for trajectory analysis.

### 2.3. Processing Techniques

Most previously proposed models for image processing, that is, location, classification, or detection of ships, have focused on using handcrafted resources applied to image processing. These models are built with the expert knowledge of designers. Within the scope of handcraft features models, it is possible to point out several works that employ different techniques, such as Gabor filter in [36], for automatic target detection, discrete cosine transform (DCT) in [37] for maritime surveillance on non-stationary surface platforms, as well as Haar–Cascade [38], scale-invariant feature transform (SIFT) [39], local binary pattern (LBP) [40], support vector machine (SVM) [41], and histograms of oriented gradients (HOG) [42] for the remote sensing of ships.

As a result, the extracted features reflect the limited aspects of the problem, generating a low response accuracy of the models and a low generalization. Thus, deep learning in the computer vision research community, such as CNNs proved to be more suitable for developing and training resource extractors [43].

The techniques based on CNN dominate the most recent works, as shown in Table 1, which details the evolution of the works over the years, pointing out aspects such as the type of image used, applications, and techniques involved in each of the works. They won great strength after winning the ImageNet challenge in 2012 and have been achieving excellent results in several image processing tasks for obtaining visual information [44].

Another point that collaborates with this type of network is the evolution of the sizes of the available datasets, given that CNNs usually require a large number of training samples. With this, the use of detection models based on CNNs has accelerated even more because, according to [45], a good object detector should improve when given more training data.

**Figure 3:**
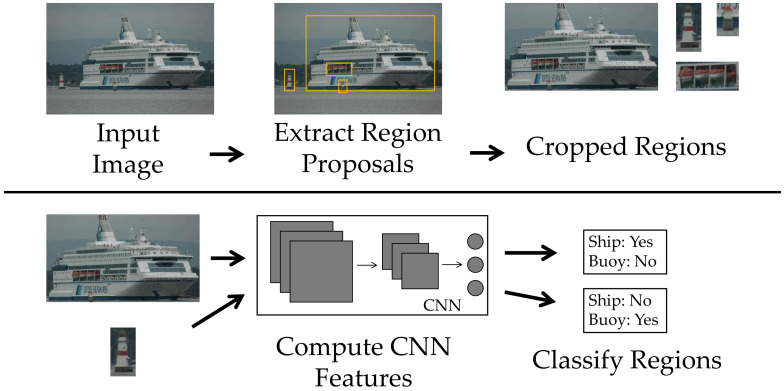
Regions with CNN features.

Within these networks, there is a subclass, the region-based convolutional neural network (R-CNNs), whose working principle is based on a selective search for object detection, generating region proposals, as shown in Figure 3. Work related to this type of technique began with the R-CNN, proposed by Ross Girshick [46]. Since then, other variations have been proposed, such as fast R-CNN [45], faster R-CNN [47], mask R-CNN [48], single-shot detector (SSD) [49], YOLO [50], YOLOv2/9000 [51], YOLOv3 [52], YOLOv4 [52], and YOLOv5 [52]. These models have some modifications in their topologies to increase their speed and prediction performances or even to add a new function, as is the case of segmentation in mask R-CNN.

#### 2.3.1. R-CNN

It emerged with the task of localizing objects through a CNN that could have high detection capability even with a small amount of annotated samples for its training. It is basically divided into three modules. The first is responsible for generating several region proposals without a specific category, by a method called selective search (SS) [53]. The second is an CNN, which extracts a fixed number of features for each of the proposals. Finally, the third module is based on a linear SVMs trained specifically for each possible class. With this, this network can not only locate the object, but also inform which of the possible classes it belongs to. This classification is performed through a score generated by the [46] classifiers.

#### 2.3.2. Fast R-CNN

Fast R-CNN introduces single-stage training with an update of all layers and avoids disk storage for feature caching [45]. Regarding the detection task, it has the advantage of achieving higher mean average precision (mAP) compared to its standard version. In this model, the linear SVMs used in R-CNN is replaced by a softmax classifier. Using the same training algorithm and hyperparameters used in R-CNN, they train a new SVM to be the classifier for fast R-CNN and justify the use of softmax by achieving a slight advantage in mAP over it [45].

#### 2.3.3. Faster R-CNN

This model uses the region proposal network (RPN), which comprises CNNs capable of providing region proposals to fast R-CNN, informing at the same time the object boundaries and the scores of each proposed region. RPN calculates proposal regions much faster and more efficiently compared to SS. Moreover, it brings another advantage by sharing convolutional layers between the proposal generation network and the classification network, optimizing the network training [47].

#### 2.3.4. Mask R-CNN

It follows the same principle as faster R-CNN but has a second output in the model for segmenting objects [48]. The pixel-by-pixel object segmentation is performed through the superposition of an outline, applied by this second output. This overlay mask is applied to each region of interest (RoI) and is based on the fully connected neural network (FCNN) model [54].

**Table 1:** Models and features on related works.

	Image View		Approaches		
**Papers**	**Side View**	**Remote**	**Localization**	**Classification**	**Techniques/Models**
2017 [55]	-	x	x	-	FusionNet
2017 [56]	x	-	-	x	VGG16
2018 [57]	x	-	x	-	Faster R-CNN+ResNet
2018 [58]	-	x	x	-	ResNet-50
2018 [59]	-	x	x	-	SNN
2018 [60]	-	x	x	x	Faster R-CNN+Inception-ResNet
2018 [61]	-	x	x	-	RetinaNet
2018 [62]	-	x	x	x	R-CNN
2018 [63]	-	x	x	-	R-CNN
2019 [64]	-	x	-	x	VGG19
2019 [65]	-	x	-	x	VGG16
2019 [66]	x	-	-	x	Skip-ENet
2019 [67]	-	x	x	x	Cascade R-CNN+B2RB
2019 [68]	-	x	-	x	ResNet-34
2019 [69]	x	-	x	-	YOLOv3
2019 [70]	-	x	x	x	VGG16
2019 [71]	x	-	x	-	Faster R-CNN
2020 [72]	-	x	x	x	SSS-Net
2020 [73]	-	x	x	x	YOLOv3
2020 [74]	-	x	x	x	CNN
2020 [75]	x	-	x	-	CNN Segmentation
2020 [76]	-	x	x	-	YOLO
2020 [77]	-	x	x	x	ResNet-50+RNP
2020 [78]	x	-	-	x	CNN
2020 [79]	x	-	x	x	YOLOv4
2020 [80]	-	x	x	-	YOLOv3
2020 [81]	-	x	-	x	VGG16
2020 [82]	x	-	x	-	Mask R-CNN+YOLOv1
2021 [83]	-	x	x	x	Mask RPN+DenseNet
2021 [84]	-	x	x	-	VGG16
2021 [85]	x	-	x	x	SSD MobileNetV2
2021 [86]	x	-	x	x	YOLOv3
2021 [87]	x	-	x	-	Faster R-CNN
2021 [88]	x	-	x	-	R-CNN
2021 [89]	x	-	x	x	BLS
2021 [90]	x	-	x	-	YOLOv5
2021 [3]	x	-	x	x	MobileNet+YOLOv4
2021 [91]	-	x	x	x	Cascade R-CNN
2021 [92]	x	-	x	x	YOLOv3
2021 [93]	-	x	x	x	YOLOv3
2021 [94]	x	-	x	x	YOLOv3
2021 [95]	-	x	x	x	YOLOv4
2021 [96]	x	-	x	x	ResNet-152
2021 [97]	-	x	x	-	Faster R-CNN
2022 [92]	x	-	x	x	YOLOv4
2022 [98]	-	x	x	-	YOLOv3
2022 [99]	x	-	x	x	MobileNetV2+YOLOv4
2022 [100]	-	x	x	x	YOLOv5

#### 2.3.5. SSD

Compared to previous methods that take two stages, SSD is a more straightforward method because it encapsulates all computations in a single deep neural net, eliminating the need to generate object proposals in multiple stages. This increases the speed of the system and facilitates training by providing a unified structure for training and inference. It scores bounding boxes and adjusts to best match the shape of the object and uses boxes of different proportions to handle objects of different sizes [49].

#### 2.3.6. YOLO

Like SSD, this is also a single-stage detector, which can have its optimized performance within its unified detection model. In this method, object detection is performed as a regression task for bounding boxes, which, at the same time, provides the object locations with their respective classes. The primary source of error in this network is in the incorrect location of small objects [50].

## 3. Datasets

Datasets are structured collections of data that are used by computer vision models during their training and validation stages. Different datasets have been created throughout the literature for visual tasks.

Image databases, in general, whether for ship classification or other purposes, usually have their images divided into classes. The number of images, the number of classes, and the complexity of visual separation of these objects directly affect the training and the results of computer vision systems.

Some datasets have a considerable imbalance concerning the number of images in each class, or even very similar classes. In datasets with many classes, many training iterations may be necessary to achieve good accuracy and other parameters related to system accuracy, such as precision, recall, and F1-score. Even in datasets with few classes, if the similarity between the objects of the two classes is high, it can also require a large number of iterations [101].

In the case of the maritime scenario, for example, architectures generally make many more mistakes when relating classes to ships than when differentiating a ship from a buoy or even some piece of wood, metal, or rubber lost at sea. This occurs because the ability to visually separate different categories of objects depends on the similarity between them in the image classification process. Therefore, some categories are more difficult to distinguish than others [102].

To suppress this problem, some models also create classes of mountains, trees, buildings, sky, and pier objects, to minimize false ship detection. However, to avoid high pollution of output result elements, some of these networks do not visually deliver the markup of these classes [7].

As long as the images are well curated, the detection and classification performance tends to increase with the expansion of the training data, as long as it is of good quality. For this reason, increasingly more extensive databases are being built, such as the the MARVEL dataset [103], which has more than 2 million images.

Considering general-purpose datasets, it is possible to cite MS COCO [104], CIFAR-10 [105], PASCAL VOC [106], OpenImage [107], and ImageNet [108] as some of the most used datasets containing ship images [109]. These datasets contain thousands and even millions of images divided into different classes, which serve as the basis for the training and validation of object detection and classification models. Each of these datasets contains a class of generic ships. According to Table 2, it is possible to obtain 11,570 images with the sum of the samples of these datasets.

However, specialized ship datasets have many more images of ships and subdivide these ships into sub-classes, giving more detail to the identified object. Table 3 lists some of these specialized datasets, providing the number of classes, the number of images of the ships, and the spatial view of these ships, where the photo datasets are divided into two groups: photos taken from the sides of the ships, in any angle within the 360° of the ship, or photos taken from the top of the ship, usually captured by satellites and classified as remote.

### 3.1. Dataset Diversity

**Figure 4:**
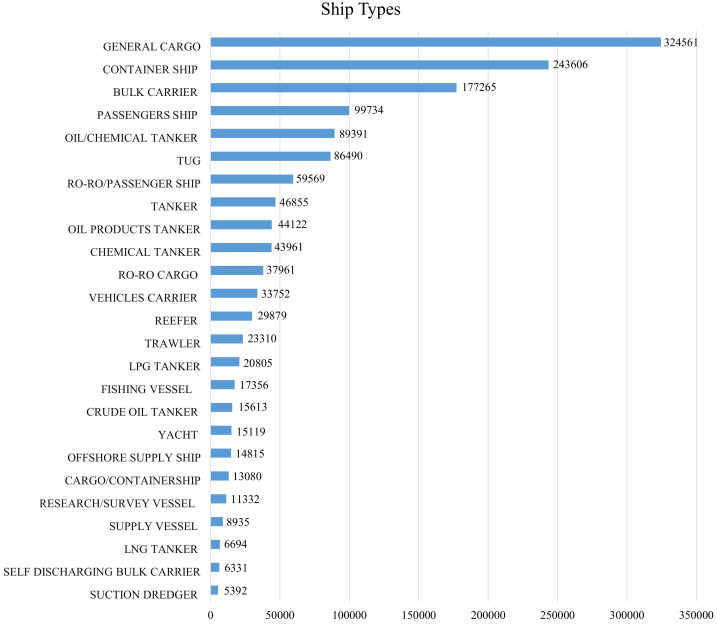
Samples by classes in the MARVEL Dataset.

**Figure 5:**
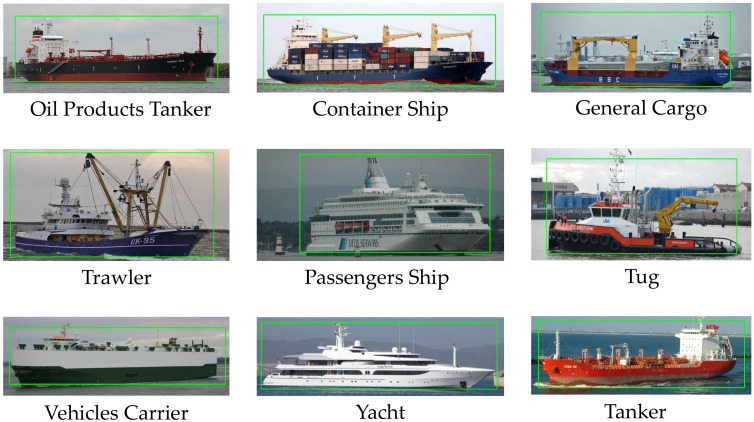
Examples of classes in MARVEL dataset.

Going into the diversity of datasets, when analyzing the MARVEL dataset, as shown in Figure 4, there is a significant imbalance between the number of samples in each class, as this is a natural reflection of a realistic environment, where there are many more ships of one type than of another. In addition, some ship types are pretty similar in size and shape, while others are remarkably different, as shown in Figure 5, which can lead to a better classification between very distinct classes and not so good for similar ones. Finally, there are also pose, brightness, background clutter and scale variations, which can negatively influence the visual system performance.

The diversity of a dataset is based on the visual variation of its samples. In the case of ship images, the most common differences between samples are variations in background, scale, position, illumination, quality, size, viewpoint, and possible occlusions. These variations can be caused by several elements, such as the distance and position chosen for capturing the photo, the capture devices themselves, and the climatic and environmental conditions.

The detection and classification models must maintain a certain sensitivity to these differences, providing stable results, even with the complexities found in maritime environments. Therefore, the data used for training and validation of image processing architectures must have diversity so that the architectures can adapt to all these influences during training [112].

#### 3.1.1. Background and Lighting

The information present in an image is used in the most diverse computer vision tasks. When considering, for example, face recognition, the separation between a front face and the background is easily performed by a background subtraction algorithm and generates a low computational cost due to the standard geometric shape of the face [117].

In the case of ships, there is a greater diversity in formats, sometimes even within the same class. This means that a single ship often can have more than one tag since its characteristics become confused with those of the environment. To solve this problem, there exist some techniques, such as the non-maximum suppression algorithm, that help avoid excessive tags by eliminating overlapping regions [118].

Another factor that can mainly compromise the detection stage is the lighting present in the images, which can sometimes cause objects in the scene to be mistaken as part of the ships and thus generate problems during the training.

#### 3.1.2. Scale and Spatial Vision

During training, the images of the ships collected have their characteristics and patterns used to build models that will make the classification process. Therefore, the scale of the ships within the image is of the utmost importance, as tiny images can contain a limited richness of detail.

**Figure 6:**
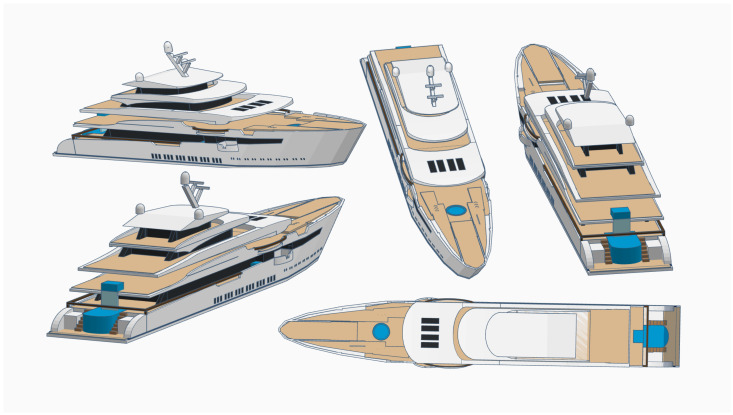
Different types of ship views.

Generally, the datasets and applications are separated into two classes of viewpoints: those that work with side view images, i.e., datasets where the pictures were taken from the sides of the ships as shown in Figure 6, and remote sensing images, which work with pictures taken from the top of the ships, usually with images taken by satellites [7].

Regardless of the type of application, whether the side view or remote view is chosen, once the model is trained with images of greater diversity, it is able to better generalize each of the classes, becoming more capable for use in a real scenario. If the system is to identify ships always from the same point of view, the choice of training samples always in the same position may be better to obtain good results.

#### 3.1.3. Size, Quality, and Resolution

Generally, most models benefit from the larger size, quality, and resolution of images when the application does not involve storage, time, or processing power. This is because when a model receives higher quality images, it is able to extract more characteristics from the objects in it, which consequently can positively influence the assertiveness of the architecture.

**Figure 7:**
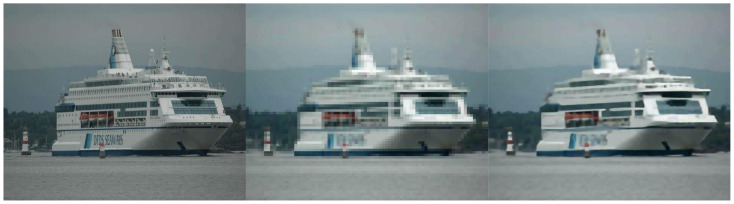
Comparison of different image resolutions.

Images with low pixel count, or even blurred images, such as Figure 7, may cause the system to be unable to extract the characteristics necessary to separate the classes. Consequently, the resulting architectures may have a degree of assertiveness lower than expected. This is usually the reason for applying the preprocessing step to the images before the detection or classification process [9].

#### 3.1.4. Occlusion and Position

**Figure 8:**
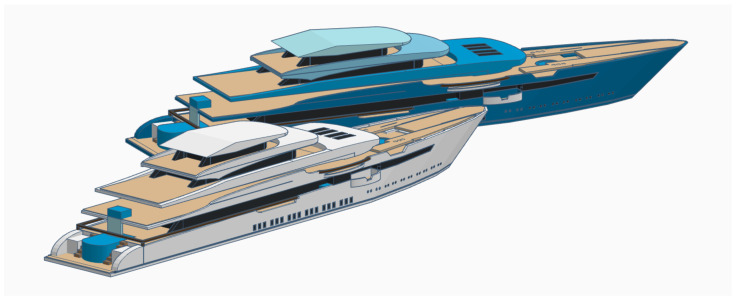
Example of occlusion.

Because the image collection is performed both offshore, in harbors or at satellites, a ship may appear partially within the image. This can be caused by the ship or by occlusions, which might be caused by other objects or even by other ships, shown in Figure 8.

Thus, according to the authors of [112], one should not ignore occlusion. Instead, it should be considered so that the trained model handles the occlusions presented in the validation step. At the same time, some care must be taken so that the position of the ship within the frame still preserves features that contribute to the training. Similarly, the partial occlusion of some objects must also preserve features of the original ship. Otherwise, these samples can directly interfere with an architecture’s ability to perform good training.

#### 3.1.5. Annotations and Labels

In machine learning and deep learning dataset, an annotation is a file that contains some data information referring to the image. The primary information stored in this file are the coordinates of the object’s spatial position within the image and the class to which the object belongs [119].

The labeling process can be carried out using annotation tools, either manually or with some automatic processing. When annotating an image, each image’s metadata are added to the dataset. Some of the datasets used in the literature already have annotations that can be used during the model training stage [112].

These annotations are essential because they allow models to understand where an object is positioned within a given image and its classification, so both detection and classification models can use these data as a reference when adjusting their weights during the training phase. With this, the model considers only the area of interest in the image. Then, after the model is trained on the labeled images, it later uses these training weights to identify these classes in new, previously unseen images.

**Figure 9:**
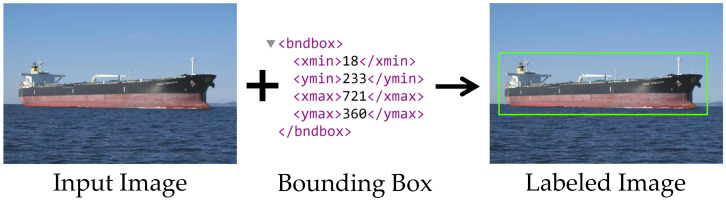
Example of a bounding box.

These annotations usually come in separate files that accompany the images. However, the simplest ones are generated with just the four boundary points, which are used to build the box that marks the position of the ship, as shown in Figure 9. With this, the models have access to the object’s position within the image and to which class it belongs. Either detection models or simply direct classification models can use these data as a reference when adjusting weights during training. This enables the model to identify the parts of interest in the image. Once the model is trained with the labeled images, it uses these training data to later identify these classes in new and previously unseen images.

The annotation files accompanying the images are usually separated in another format, such as “.xml”. The simplest ones come with four positions, which are the boundaries used to construct the box that marks the position of the ship, as shown in Figure 9. Some more complex annotations may contain multilevel classifications, segmentation data, and multiple object tags.

Along with the four coordinates, usually, the class to which the ship belongs to is also described so that the system can use these data when building the model. Files with more complex annotations can also bring climate, relative humidity, latitude, and longitude positions of the ship. However, most detection and classification systems disregard that information and only need the object’s classes and bounding boxes to perform the training.

Annotations enrich the information about the object in the image so that the detection model is able to learn from this information. To this end, numerous annotation software programs are widely used as crucial tools for preparing images for training. These tools were developed because of the increasing demand for training data and are widely employed.

Table 4 shows a comparison of the tools, which are divided into categories. The working environments being local, those tools that require the software to be installed on a machine, and browser, those that can be only used through a web-browser. The tools can be used either in online or offline modes, as shown in the Table 4. Each tool has different characteristics, such as the processing data that are the input data that the tool annotates, e.g., images and videos, and can even support 3D point cloud annotations, commonly used by radio detection and ranging (RADAR) and light detection and ranging (LiDAR) sensors.

Another essential feature is that these tools offer different types of annotations, being the most used polygons and rectangles. However, some tools can even offer brushes and pencils to draw each object differently. Each tool offers different file formats for saving, that is, the format in which the annotations for each image or video will be saved to be used during training.

Semi-automatic labeling tools delimit objects in an image or video using a pre-trained detection model. The advantage obtained in this process is the time savings compared to manual labeling. The result is a pre-labeled set of images, which allows the user to perform subsequent tasks, such as checking and correcting labels already created or even training new models with the semi-automatically labeled samples. Table 4 compares different labeling tools that use manual and semi-automatic methods, as well as describing the operating characteristics of the different approaches.

The availability in the table refers to either paid or free tools. Furthermore, some remarks can help choose the labeling tool, such as the online support service that some offer.

In the works involving the classification task, the ships are divided into classes, whether directly applied to classify an image or even after a localization. However, within the area of ships monitoring, there are few specific standards and regulations for autonomous marine systems, which already use this detection technology with sensor systems [43]. There are some agencies to assist in the creation, regulation, and control of these systems, such as the International Organization for Standardization (ISO), International Maritime Organization (IMO), International Association for Marine Electronics Companies (CIRM) International Association of Classification Societies (IACS), International Electrotechnical Commission (IEC), International Association of Marine Aids to Navigation and Lighthouse Authorities (IALA), European GNSS Agency (GSA), International Telecommunication Union (ITU) and various classification societies themselves [43].

As the control agencies have not yet created definitive standards, each author follows the class division that best suits their work. Even though they still do not follow a pattern, some of these classes are found more frequently than others in the datasets of related works, as shown in Table 5. In this table, the works that use the separation of ships into classes are presented with their respective classes. The amount of images divided for training and testing in each of the works is also presented.

## 4. Challenges and Issues

This section is established based on proposals for future work from a review of the literature as well as the related works already mentioned above. Among the main problems, challenges, and research opportunities cited are those related to datasets, image processing techniques, data fusion, and practical applications. In addition, some recent works, such as [81], also point to some of these problems, which will be discussed throughout this chapter.

### 4.1. Datasets

Datasets represent an important part in the construction of object location, classification and detection models. In vessel datasets, it is possible to find problems common to other datasts used in automatic target recognition (ATR) problems, such as overlapping objects. However, other problems encountered, such as the high similarity between different classes of vessels, deterioration of ships and a great variety of models for each class, are inherent problems, or even more common in maritime environments than in computer vision problems in general.

There is some difficulty in accurately finding the object when the image has a considerable background complexity [77]. In [5], a maritime ship tracker is proposed, but the authors state that the proposed tracker can only work in certain weather conditions and only for some types of ships. In [135], the authors also state that the presented technique shows errors in specific scenarios where the sea color is drastically changed or when the horizon line suffers partial occlusion by other objects.

Based on these problems, the authors of [136] make a practical study of detection with several architectures, such as faster R-CNN, YOLOv2, and YOLOv3 in datasets of images with weather and lighting interference to evaluate the accuracy of the models. In [137], the authors explain that images related to the maritime scene suffer several influences related to weather and lighting factors, resulting in unclear targets in the image.

The proposed solution presented in the study is to attack the problem on three fronts, improving the image acquisition hardware technology, creating an image preprocessing step, and increasing the dataset used for training, with images that have multiple targets and high diversity [137]. Regarding the issue of diversity, the use of datasets in the CNN models must have good image quality and also represent the shapes of the objects, which are taken from multiple sides [78].

Many authors point out that the lack of substantially extensive datasets hinders the construction of their models. The search for high-quality datasets is a shared objective that ranges from authors who develop simpler models to those who seek to validate their systems in more complex environments. For example, the authors of [39] claimed that the popularization of high-resolution remote sensing data could make the proposed method widely applicable. In [65], the authors claimed that they will compare their proposed model with state-of-the-art results while expanding the datasets. In [138], the authors also stated that they will make efforts to expand the dataset to try to obtain a robust detection of the system. Even some work on more recent detector enhancements, such as the YOLOv5, still points out that they intend to perform retraining on large datasets to evaluate the new results [90].

Some works, such as [42], point to good results for the task of automatically locating and recognizing coastal ships in remote sensing images of large scenes. However, they state that they have their efforts in the development of new multimodel methods capable of recognizing more types of ships and that for this, it is necessary to obtain samples of other classes of ships. In this search for an increase in the number of classes capable of being recognized by the models, another problem faced and reported by other works, including those focused on new datasets, is the imbalance of samples in each class. This can be easily seen when when internally analyzing the structure of large databases such as MARVEL, for example [103]. Thus, some authors, such as [83], reaffirm this problem, citing that the efforts of their works have been to reduce the class imbalance, feeding the less favored classes with more samples, thereby decreasing the risk of overfitting that can be caused by the imbalance during the training of the models. The authors of [70] also pointed out the risks of overfitting when the dataset contains many small or poor-quality images.

As an alternative to the difficulties presented by the authors regarding limited databases, adding bad images to the model, or even the imbalance between classes, Ref. [68] suggested leveraging synthetically generated images to compose the training data since they reinforced the idea that CNNs outperform classical object recognition methods when provided with enough data for good training. In their study, they demonstrated that the same ship classifier trained on a bank of real-only images performs worse compared to the same classifier trained on that same dataset with the addition of synthetic images [68].

A second alternative to the limitation posed by the dataset would be transfer learning. Transfer learning is a machine learning technique that stores the knowledge gained from solving a problem and applying it to a different but minimally related problem [139]. For example, Ref. [140] presents the application of this type of technique in a ship recognition task in infrared images. With this, even if there is an imbalance between the samples of each class, it is possible to improve the model’s performance. The work presented in [64], which uses visible light remote sensing images, also suggests that transfer learning solves the limitation of the number of images on datasets and improves the convergence speed of the model.

Finally, the last challenge pointed out by the authors in the context of the dataset is about images annotated with precise bounding boxes to provide an effective and available database for training and validation. The idea is to try to reduce as much as possible the images that are in the wrong classes or with no boat present, called negatives. With that, the result of the complete training or transfer learning tends to improve even more [110]. Therefore, a second proposal made in [111] is, instead of discarding negative images, using them during training to explore the effect of these samples within the system in order to develop a more robust algorithm.

### 4.2. Image Processing Techniques

The techniques chosen to build object localization, classification, and detection systems are another essential part of the system through which the received images are converted into information. In the tests performed in [141], a large dataset of images captured by several optical satellite sensors was submitted to a hierarchical classification architecture, which was able to eliminate candidate regions belonging to objects that did not represent ships. Moreover, other hierarchical classification techniques can be applied with newer architectures. The authors admit the need to improve the model or refine the training parameters to improve the detector. This would be another way to mitigate problems related to complex backgrounds in the received images, now improving the technique instead of dataset changes.

When discussing improvements improvements in detectors and classifiers, it is also possible to find several works that advocate this idea, such as [63,89], which followed the line of improving or adjusting parameters in the model to increase accuracy, instead of improving with the evolution of the dataset only. In [142], the authors advocated increasing the dataset and the number of classes provided for training. Another objective is to adapt the detection models by changing their parameters and the architectures themselves to compare their results to the original ones and verify the accuracy increase.

Another work that aims for future improvements by improving the architecture is the one presented in [143], where the authors used the SSD detection techniques, aiming for automation in the container terminal. In [86], the authors also relied on the optimization of existing methods, where the YOLOv3 architecture is optimized to detect ships at a higher frame rate without sacrificing detection loss. Other work also has as future tasks the optimization of the ship target recognition capability so that the entire model performance can be further improved [74]. The experiment data are part of public Google Earth data and commercial satellite imagery [74]. To select these parameters that optimize the system, Ref. [144] stated that the empirical way can work very well, but stated that a more systematic way to select these parameters can be a target of future research.

Furthermore, regarding the techniques, there are some recent works such as [145,146], which operate on a sky-sea basis, i.e., using the dividing line between sea and water to help locate the ship, and have interesting effectiveness for open sea applications. The work [147] suggests research that combines CNNs with handcrafted techniques to perform a sea–land separation, decreasing the application restriction of these systems. The work [148] also intends to deal with the problem of low contrast that sometimes occurs in a dynamic ocean scenario, which generates waves with a different reflected color tone than expected in a pixel analyzed in the image where the ocean is present.

Among the works that present possibilities for combining techniques for future research, there is the case of [149], which introduced a method to exclude confusing samples and thereby reduce the problem of overlapping classes. Future work in this paper aims to integrate this method with other techniques. This is also the research theme in [81], but instead of using features from other models in their own, the authors performed the inverse process. They proposed two feature representation schemes that can be incorporated into most CNN models and bring an increase in the classification performance of the models, taking advantage of the possibility of end-to-end training. The work basis in a new benchmark and an attribute guided multilevel feature representation network for fine-grained ship classification in optical remote sensing image [81].

Besides the combination of multiple techniques in a single architecture, it is also possible to find works that point to future tasks, the inclusion of preprocessing models. These tasks range from simpler challenges, such as cropping, aligning and resizing images [115], to even more complex ones, such as noise removal [150,151,152] and image super-resolution systems [12,16]. Enhanced images, generated by these types of techniques, increase successful detection and reduce false detection [153]. Based on that, Ref. [154] suggested as future work the use of super-resolution GAN models to improve image quality and thus be able to identify attributes over long distances.

The performance of a ship ATR system is not only defined by the chosen algorithm, but also by the image quality and the result generated by the feature extraction technique. By improving the processing model and the image quality, the target recognition rate will be improved [155]. For example, [66] proposes that in the future, their segmentation-based model be used by another one for image preprocessing so that the final architecture can be used in different weather or light conditions. In parallel, the authors also hope to incorporate distance estimation algorithms to contribute to research on autonomous surface vehicles. Similarly, Ref. [156] also aims to develop detection models for target tracking.

### 4.3. Data Fusion

Within the literature, there are some works, such as [43], which already explore the situational awareness field. In this type of approach, the optical sensors do not act in isolation, and there is a fusion of data with other sources of information, which allows the generation of a positioning map of ships in an autonomous way.

For this map to be generated, there is a global effort regarding the creation of regulations and standards which allow the reliability and integrity of the information generated. Furthermore, this type of approach aims to design an artificial intelligence (AI) algorithm capable of merging the different types of sensors and information sources. The idea of this fusion is to implement a system capable of obtaining a positioning inference of less than 3 m, as well as collaborating for autonomous navigation [43].

The current availability of sensors capable of collecting information at different levels of an object allows observations made from different acquisition sources to be combined to obtain a more detailed description of the scene. For example, Ref. [157] presents as a focus of future studies the increase in the robustness of the system through multispectral remote sensing, aiming to identify ships that are close to land.

Each of the sensor types has its advantages and disadvantages. Among them, it is possible to mention costs, dependence on the angle of the observation aspect, variation of resolution with distance, susceptibility to atmospheric influences [158]. The differences and advances in the different types of sensors mean that research into new image processing methods and tools never stands still. Just as each data source can generate unique information, combining features generated by these different sources makes sense. The systems developed for this purpose propose the use of fusion techniques during this processing chain in order to obtain at the end a situational awareness of the region or maritime object under analysis [158].

Some works in the literature already bring some of these fusion proposals. For example, in [159], the authors create an autonomous collision avoidance system using a fusion of different sensor sources as global positioning system (GPS), RADAR, LiDAR, automatic identification system (AIS), and optical sensors, but say that more studies are needed in various conditions of real maritime traffic to verify stability and robustness.

In [109], the authors also intend to evaluate the benefits of autonomous navigation and the improvement of navigation safety through tracking techniques. In this line, Ref. [160] reinforces that some more handcrafted image processing methods can reduce computational costs during tracking, that is, the continuous observation of navigation. In [60], the focus of future work is on the use of data involving AIS. In [161], the authors propose the combination of radar information in addition to AIS and tracking to evaluate suspicious activities within the maritime scenario. The work [162] also considers the integration of the detection method with RADAR and AIS systems for future implementations.

From more recent works, such as [43], to works more than a decade old, such as [163], they all have the exploration of complete surveillance solutions as a common point. Whether they are based on electro-optical and infrared sensors, with the use of multiple image processing techniques, or works that bring together data from various families of sensors, the ultimate focus of the system should be to support the fusion of information to interpret scene activity, associate targets with the offense committed or the threat they correspond to, and generate situational information to a control center [163].

### 4.4. Practical Applications

Within the entire literature review, several works cited that they aim at the practical implementation of their systems to provide the collection of visual information in the maritime scenario [164]. For this to be possible, it is necessary to evaluate several variables, and computational capacity is usually one of the most important deciding factors when evaluating the feasibility of practical implementations. For this reason, some works that aim to create computer vision systems that run in real time, such as [165], make several analyses related to processing time and the use of devices with different computational capacities.

Some authors, such as [157], conclude that their system responds more efficiently if their candidate region search method is applied offline. However, the algorithm must be employed to create a real-time situational view. Therefore, papers such as [166], which demonstrate operation only for still images, suggest using it on continuous video streams. In a review in [110], the authors suggest performing parallel data collection in addition to processing. They claim that to develop systems for autonomous surface ships, datasets with images collected and annotated from cameras installed on moving ships are needed. With this, the dataset used to train the computer vision model will be extremely close to the maritime scenario in which the autonomous ship will navigate, making the system more robust than if the model only trained it with images of ships docked in ports.

In [56], the authors say that the architecture adopted there can be applied to real-time computer vision problems by installing cameras at harbors. They also say that systems will store the images collected during operation to retrain the system, making it more robust with samples of different degrees of illumination captured throughout the day.

When the scenario of practical implementations is explored, it is possible to find works where an embedded target detection system was implemented using the YOLOv4 architecture [79]. However, the authors themselves say that there is room for further improvement in the detection rate in the experiment and that they intend to retrain the same system with more data and other categories to achieve better results.

Finally, the authors of [6] propose and implement an object detection algorithm for maritime surveillance, where low processing power embedded systems are the focus of the application. The processing architecture was built to reduce the volume of data processed in maritime surveillance systems. In this work, future research directions focus on designing lighter weight detection architectures to achieve good performance, even on computationally limited devices.

## 5. Conclusions and Future Work

This paper presents a review of ship localization, classification, and detection methods based on optical sensors. This literature review made it possible to find the main challenges and open problems, besides exploring the techniques and architectures used by several authors.

It is possible to state that CNNs have been explored with greater intensity over the years regarding processing techniques. It is possible to recognize the advancement of this type of technique with the observation of high-precision architectures, with the ability to detect small objects, even in scenarios with noise and other sources of interference. Moreover, the evolution of the computational capacity of devices allows these techniques to be employed in practical applications, replacing the need for human intervention in several tasks [88].

Still exploring the techniques and models already devised, several detection algorithms and practical maritime decision-making systems should be applied to the same dataset to evaluate all works with the same metrics and datasets, just as several face verification works use labeled faces in the wild (LFW) as a standard benchmark [167]. As in [62], several authors have already said that they intend to verify this scalability of the technique on other datasets. This idea is indeed valid since each of the datasets brings some particularities that are new challenges for the model.

Research within datasets has shown that there is no uniformity in their use. Each author works with their database. Therefore, more effort is needed to create large-scale datasets that are readily available, so that the community can begin to have a more reliable standard of comparison. About labeling, many authors claim to have done it manually. Therefore, the ideal would be to explore CNNs that could be adapted to generate the bounding boxes or pixel-by-pixel labeling automatically or semi-automatically.

The combination of deep learning and navigation data has the potential to solve maritime situational awareness problems. This task is quite challenging but of equal or greater importance for applications in maritime environments. The situational awareness of all objects present can bring many benefits to any system. Different architectures are proposed to improve the ability of automatic target recognition and search, each with its advantages and disadvantages.

The research involving practical implementations is mainly based on the fact that all the extra tasks of localization, simple or hierarchical classification, a combination of detection techniques, preprocessing, AIS, and data fusion may increase the amount of input data and results in an additional computational cost. From this arises the focus of analyzing these computational costs based on the available technological conditions.

Therefore, it is possible to conclude that all research efforts within the literature review fall within the following four research lines:

1 Creating large-scale fine-grained datasets with higher diversity and already labeled samples, using synthetic data, and improving the balance between classes.
2 Creating, optimizing, and combining image processing techniques, including preprocessing and the use of transfer learning or similar techniques.
3 Usage of different sensors and data sources to operate in conjunction with the optical sensors, thereby generating a situational awareness of the monitored maritime region.
4 Practical analysis of the systems, indicating their performance and speed in real scenarios, where the complexity may be higher than in the datasets.

With this, it is possible to conclude that this paper describes the main open problems pointed out by the literature, aiming to influence the research of new work and better delimit the challenges to be overcome.

## Figures and Tables

**Table 2:** Generic datasets.

Dataset	Ship Count
COCO [104]	3146
CIFAR-10 [105]	6000
PASCAL VOC [106]	353
OpenImage [107]	1000
ImageNet [108]	1071

**Table 3:** Remote and side view ship datasets.

Dataset	Side View	Remote	Images	Ship Classes
VAIS [110]	x	-	2865	15
ABOShips [109]	x	-	9880	9
MCShips [111]	x	-	14,709	13
Singapore [7]	x	-	17,450	6
SeaShips [112]	x	-	31,455	6
MARVEL [103]	x	-	2,000,000	29
HRSC2016 [113]	-	x	1061	19
Airbus Ship Detection [114]	-	x	208,162	1
BCCT200 [115]	-	x	800	4
ShipRSImageNet [116]	-	x	3435	50

**Table 4:** Comparison of tools for labeling images and videos.

Tools	Environment	Conectivity	Processing Data	Annotation Types	Output Data	(Semi)Automatic Labeling	Availability	Remarks
ImgLab [120]	Browser and local	On/Offline	Images	Points, circles, rectangles, and polygons.	dlib XML, dlib pts, VOC, and COCO	No support	Free	-
VoTT [121]	Browser and local	On/Offline	Images and videos	Rectangles and polygons.	CNTK, Azure, VOC, CSV, and VoTT(JSON)	Support	Free	-
CVAT [122]	Browser and local	On/Offline	Images and videos	Points, lines, cuboids, rectangles, and polygons.	VOC, COCO, etc.	Support	Free	-
Labelimg [123]	Local	Offline	Images	Rectangles.	VOC, YOLO, and CSV.	No support	Free	-
Labelme [124]	Local	Offline	Images and videos	Points, circles, lines, rectangles, and polygons.	VOC, COCO, etc.	No support	Free	-
VGG Image Annotator (VIA) [125]	Browser	On/Offline	Images, videos, and audios	Points, circles, lines, ellipses, rectangles, and polygons.	VOC, COCO, and CSV	No support	Free	-
SuperAnnotate [126]	Browser and local	On/Offline	Images, videos, and texts	Points, lines, ellipses, cuboids, rectangles, polygons, and brushes.	JSON and COCO	Support	Paid	Online support
Supervisely [127]	Browser and local	On/Offline	Images, videos, and 3d point cloud	Points, lines, rectangles, polygons, and brushes.	JSON	Support	Paid	Online support
MakeSense [128]	Browser	Online	Images	Points, lines, rectangles, and polygons.	YOLO, VOC, and COCO	Support	Free	-
LabelBox [129]	Browser	Online	Images, videos, and text	Points, lines, rectangles, polygons, and brushes.	JSON and CSV	Support	Paid	Online support
DarkLabel [130]	Local	Offline	Images and videos	Rectangles.	VOC and YOLO	Support	Free	Online support
Autoannotation [131]	Browser	On/offline	Images	Rectangles.	YOLO	Support	Free	-

**Table 5:** Class division in related works.

Papers	Type	Classes	Train	Test
2008 [132]	2	Aircraft Carrier and Destroyer	-	270
2009 [133]	4	Carrier, Cruiser, Destroyer and Frigate	-	98
2010 [134]	4	Ark Royal, Arizona, Arleigh and Connelly	-	32
2017 [42]	12	Military Ships (Aircraft Carrier, Submarine, San Antonio, Arleigh Burke, Whidbey Island)	200	80
2017 [56]	5	Containers, Fishing Boats, Guards, Tankers, Warships	-	300
2018 [60]	9	Passenger Ship, Leisure Boat, Sailing Boat, Service Vessel, Fishing Boat, Warship, Generic Cargo Ship, Container Carrier and Tanker.	30000	20
2018 [62]	3	Cargo Ship, Cruise and Yacht	-	-
2019 [68]	4	Barge, Cargo, Container and Tanker	-	-
2019 [64]	3	Oil Tankers, Bulk Carriers and Container Ships	-	-
2020 [77]	7	Aircraft Carrier, Destroyer, Cruiser, Cargo Ship, Medical Ship, Cruise Ship and Transport Ship.	24	6
2020 [73]	3	Passenger Ships, General Cargo Ships and Container Ships	-	-
2020 [74]	4	Destroyers, One Bulk Barrier, Submarine and Two Aircraft Carriers	-	500
2020 [78]	2	Fishing Ships and Military	398	16
2020 [81]	23	Non-ship, Aircraft Carrier, Destroyer, Landing Craft, Frigate, Amphibious Transport Dock, Cruiser, Tarawa-Class Amphibious Assault Ship, Amphibious Assault Ship, Command Ship, Submarine, Medical Ship, Combat Boat, Auxiliary Ship, Container Ship, Car Carrier, Hovercraft, Bulk Carrier, Oil Tanker, Fishing Boat, Passenger Ship, Liquefied Gas Ship and Barge	5165	825
2021 [83]	4	Warcraft, Aircraft Carrier, Merchant Ship and Submarine	-	-
2021 [86]	6	Warship, Container Ship, Cruise Ship, Yacht, Sailboat and Fishing Boat	-	-
2021 [91]	15	Aircraft Carrier, Oliver Hazard Perry Class frigate, Ticonderoga-class Cruiser, Arleigh Burke Class Destroyer, Independence-class littoral combat ship, Freedom-class littoral Combat Ship, Amphibious Assault Ship, Tanker, Container Ship, Grocery Ship, Amphibious Transport Ship, Small Military Warship, Supply Ship, Submarine and Other.	4800	1200
2021 [3]	8	Bulk Cargo Ships, Engineering Ships, Armed Ships, Refrigerated Ships, Concrete Ships, Fisheries Vessels, Container Ships and Oil Tankers	-	-

## Abbreviations

The following abbreviations are used in this manuscript:

ATR	Automatic Target Recognition
AIS	Automatic Identification System
B2RB	Bounding-Box to Rotated Bounding-Box
BLS	Broad Learning System
BN	Batch Normalization
CNN	Convolutional Neural Network
DCT	Discrete Cosine Transform
DeFMO	Deblurring and Shape Recovery of Fast Moving Objects
DeblurGAN	Deblur Generative Adversarial Network
EDSR	Enhanced Deep Super-Resolution Network
ESRGAN	Enhanced Super-Resolution Generative Adversarial Network
FPN	Feature Pyramid Network
FusionNet	Fusion Network
GAN	Generative Adversarial Network
HOG	Histograms of Oriented Gradients
IACS	International Association of Classification Societies
IALA	International Association of Marine Aids to Navigation and Lighthouse Authorities
IEC	International Electrotechnical Commission
IEEE	Institute of Electrical Electronic Engineers
IMO	International Maritime Organization
IR	Infrared
ISO	International Organization for Standardization
ITU	International Telecommunication Union
LBP	Local Binary Pattern
LFW	Labeled Faces in the Wild
LiDAR	Light Detection and Ranging
mAP	Mean Average Precision
MobileNet-DSC	Mobile Network-Depthwise Separable Convolution
MDSR	Multi-Scale Deep Super-Resolution
MLP	Multi-Layer Perceptron
MOS	Mean Opinion Score
MSE	Mean Squared Error
NIQE	Natural Image Quality Evaluator
PIRM-SR	Perceptual Image Restoration and Manipulation—Super Resolution
PSNR	Peak Signal-to-Noise Ratio
RADAR	Radio Detection And Ranging
RaGAN	Relativistic average GAN
RankSRGAN	Rank Super-Resolution Generative Adversarial Network
ResNet	Residual Network
ResBlock	Residual Block
RetinaNet	Retina Network
RNN	Recurrent Neural Network
RoI	Region of Interest
RRDB	Residual-in-Residual Dense Block
RPN	Region Proposal Network
R-CNN	Region Based Convolutional Neural Network
LiDAR	Light Detection and Ranging
SIFT	Scale-Invariant Feature Transform
Skip-ENet	Skip Efficient Neural Network
SNN	Spiking Neural Networks
SR	Super-Resolution
SRCNN	Super-Resolution Convolutional Neural Network
SRGAN	Super-Resolution Generative Adversarial Network
SS	Selective Search
SSD	Single-Shot Detector
SSIM	Structural Similarity Index Measure
SSS-Net	Single-Shot Network Structure
SRResNet	Super-Resolution Residual Network
SVM	Support Vector Machine
VGG	Visual Geometry Group
YOLO	You Only Look Once
